# Three-dimensional plasmonic Ag/TiO_2_ nanocomposite architectures on flexible substrates for visible-light photocatalytic activity

**DOI:** 10.1038/s41598-017-09401-z

**Published:** 2017-08-21

**Authors:** Zhi-Jun Zhao, Soon Hyoung Hwang, Sohee Jeon, Boyeon Hwang, Joo-Yun Jung, Jihye Lee, Sang-Hu Park, Jun-Ho Jeong

**Affiliations:** 1 School of Mechanical Engineering, Pusan National University, Busandaehak-ro 63beon-gil, Geumjeong-gu, Busan, 609-735 Republic of Korea; 2 Department of Nano Manufacturing Technology, Korea Institute of Machinery and Materials, Daejeon, 305-343 South Korea; 3 Research Institute of Advanced Materials (RIAM), Department of Materials Science and Engineering, Seoul National University, Daehak-Dong, Gwanak-Gu, Seoul, 151-744 Korea; 4 School of Electrical Engineering, Collage of Engineering, Korea University, Seoul, 02841 Republic of Korea

## Abstract

In this study, a periodic three-dimensional (3D) Ag/TiO_2_ nanocomposite architecture of nanowires was fabricated on a flexible substrate to enhance the plasmonic photocatalytic activity of the composite. Layer-by-layer nanofabrication based on nanoimprint lithography, vertical e-beam evaporation, nanotransfer, and nanowelding was applied in a new method to create different 3D Ag/TiO_2_ nanocomposite architectures. The fabricated samples were characterized by scanning electron microscopy, transmission electron microscopy, focused ion-beam imaging, X-ray photoelectron spectrometry, and UV–visible spectroscopy. The experiment indicated that the 3D nanocomposite architectures could effectively enhance photocatalytic activity in the degradation of methylene blue solution under visible light irradiation. We believe that our method is efficient and stable, which could be applied to various fields, including photocatalysis, solar energy conversion, and biotechnology.

## Introduction

With the development of modern industry, environmental pollution and energy depletion have become serious social problems that scientists worldwide are attempting to solve. Oxide semiconductors have been widely studied as photocatalysts for application in environmental protection procedures such as water disinfection^1^, bacterial inactivation^2^, and air purification^3^. Among various oxide semiconductor photocatalysts, TiO_2_ is the most compelling because of its low cost, high stability, excellent optical properties, and good degradation of toxic organic pollutants^4–6^. However, it has some major limitations: (i) Effective utilization of visible light is difficult because of the large band gap of 3.2 eV. (ii) The recombination of photogenerated electrons and holes in pure TiO_2_ occurs very quickly, which limits its photocatalytic activity. To solve these issues, noble metals such as Ag and Au can be embedded on TiO_2_ to provide photogenerated electrons and holes under visible light irradiation. For example, Sakthivel *et al*. proved and characterized the photocatalytic activity of metals (Pt, Au, and Pd) deposited on a TiO_2_ catalyst^7^. Cheng *et al*. synthesized Ag@TiO_2_ core–shell nanocomposite nanowires via a vapour-thermal method, using Ag nanowires as templates to enhance the photocatalytic activity; the core–shell nanocomposites exhibited high efficiency^8^. In addition, Zhou *et al*. proposed a facile method for the preparation of TiO_2_-coated Au nanorods and Au/Ag nanorods with core–shell nanostructures and demonstrated the effective photocatalytic activities for the degradation of organic dyes under visible light irradiation by these materials^9^. Wu *et al*. fabricated TiO_2_ nanotube arrays (TiO_2_NTs) by anodic oxidation and then assembled Ag nanoparticles on the TiO_2_NTs to form Ag/TiO_2_ NTs by a microwave-assisted chemical reduction^10^. Singh *et al*. demonstrated the synthesis of hybrid plasmonic nanostructures using Ag nanoparticle-decorated TiO_2_ nanorods by a wet chemical method; these showed highly enhanced photocatalytic activity^11^. Li *et al*. investigated the reactive oxygen species production of seven selected metal-oxide nanoparticles and their bulk counterparts under UV irradiation at 365 nm^12^. Tian *et al*. studied the mechanisms and application of plasmon-induced charge separation on TiO_2_ films loaded with Au nanoparticles^13^. Eom *et al*. investigated periodic arrays of Ag/TiO_2_ open core–shell nanowires as enhanced plasmonic photocatalytic structures by using nanoimprinting, oblique-angle evaporation, and selective electrodeposition^14^. Other studies have attempted to fabricate composites nanomaterials of noble metals and TiO_2_ via various methods to improve the photocatalytic efficiency and photo-inactivation of bacteria by the composites^15–26^. The abovementioned studies utilized the surface plasmon resonance (SPR) of noble metals formed under visible-light irradiation to generate electrons, which were transferred to the surface of TiO_2_ and electron–hole pairs spontaneously formed. In this way, the fast recombination of electrons and holes in TiO_2_ was solved to achieve a highly efficient photocatalyst. However, most studies exploited irregular nanostructures, such as metal nanoparticles, nanoclusters, and nano-heterostructures, within TiO_2_ matrices, which limited the light absorption of the surface area, weakened SPR, and inhibited the recycling of plasmonic photocatalytic nanostructures.

In this work, periodic three-dimensional (3D) nanocomposite architectures of Ag/TiO_2_ were fabricated via nanoimprint lithography, vertical evaporation, nano-transferring, and nanowelding. The fabricated nanocomposite architectures provided strong SPR effects under visible-light irradiation, thereby demonstrating photocatalytic activity. In order to demonstrate the strong SPR effect, ultraviolet–visible (UV–Vis) absorption measurements were implemented using UV-Vis spectrometry. Furthermore, the photocatalytic activities of the fabricated nanocomposite architectures under visible-light irradiation were evaluated by the degradation of a methylene blue (MB) solution. Our fabrication process of the periodic Ag/TiO_2_ nanocomposite architectures provided the following distinctive advantages: (i) They have large light-absorbing surface areas to enhance SPR effects. (ii) Using nanoimprint and E-beam evaporation permits facile, high-throughput, and convenient post-processing. (iii) Nano-transfer and nanowelding overcomes the fabrication difficulty of metal-oxide 3D nanocomposite architectures. (iv) Nanofilms with nanocomposite architectures can be recycled. We believe that the proposed method could be applied in various fields, including photocatalysis, biotechnology, and sewage purification.

## Results

### Mechanism of SPR-enhanced photocatalytic activity

**Figure 1:**
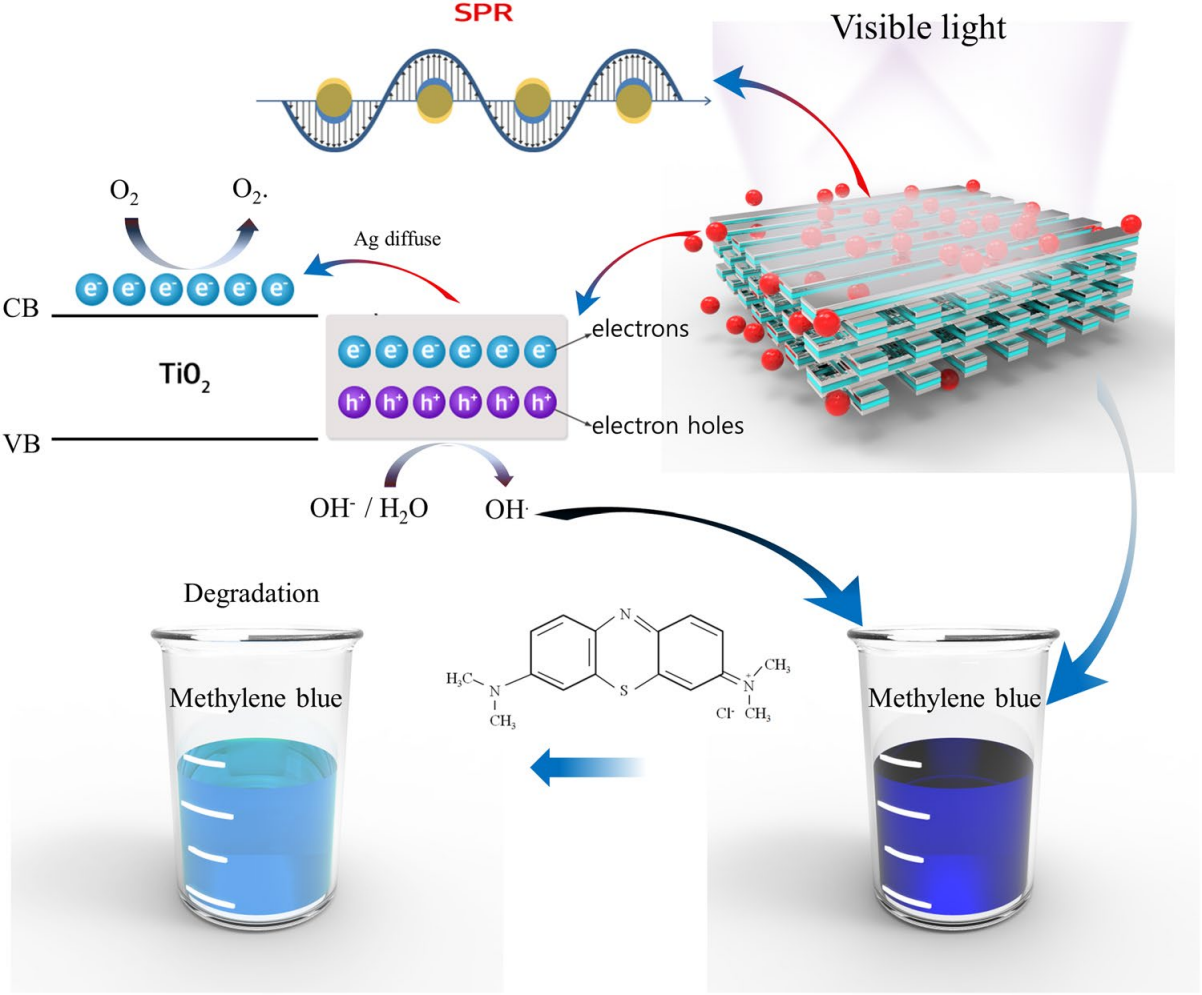
Mechanism of the Ag/TiO_2_ nanocomposite architecture. Electrons and holes in the Ag/TiO_2_ nanocomposite cross structure enhance the photocatalytic reaction by the SPR phenomenon under visible-light irradiation. Under visible light, generated electrons react with oxygen molecules to form superoxide radicals (O_2_^−∙^) and the holes in the surface of the metal layer react with hydroxyl groups to form hydroxyl radicals (OH^∙^). Superoxide and hydroxyl radicals can participate in various chemical reactions with all biological molecules, organic or inorganic.

Figure 1 shows the mechanism of SPR-enhanced photocatalytic activity on the surface of the periodic Ag/TiO_2_ nanocomposite architecture under visible-light irradiation. Under visible light, free electrons generated on the surface of the metal experience collective oscillations. When the collective oscillation frequency of the free electrons coincides with the oscillation frequency of visible light, the SPR phenomenon occurs, which enhances the generation of electron–hole pairs. The electrons generated by SPR diffuse to the surface of the TiO_2_, simultaneously generating electron–hole pairs. The generated electrons react with oxygen molecules, resulting in the formation of superoxide radicals (O_2_^−^·). The electron—hole pairs at the surface of the metal layer react with hydroxyl groups, forming hydroxyl radicals (OH∙). Both superoxide and hydroxyl radicals are active molecules and aggressive chemical substances; they can induce various chemical reactions with all biological molecules, whether organic or inorganic. Therefore, they are important in the photocatalytic degradation of organic and inorganic substances.

### Morphologies and properties of the Ag/TiO_2_ nanocomposite architectures

**Figure 2:**
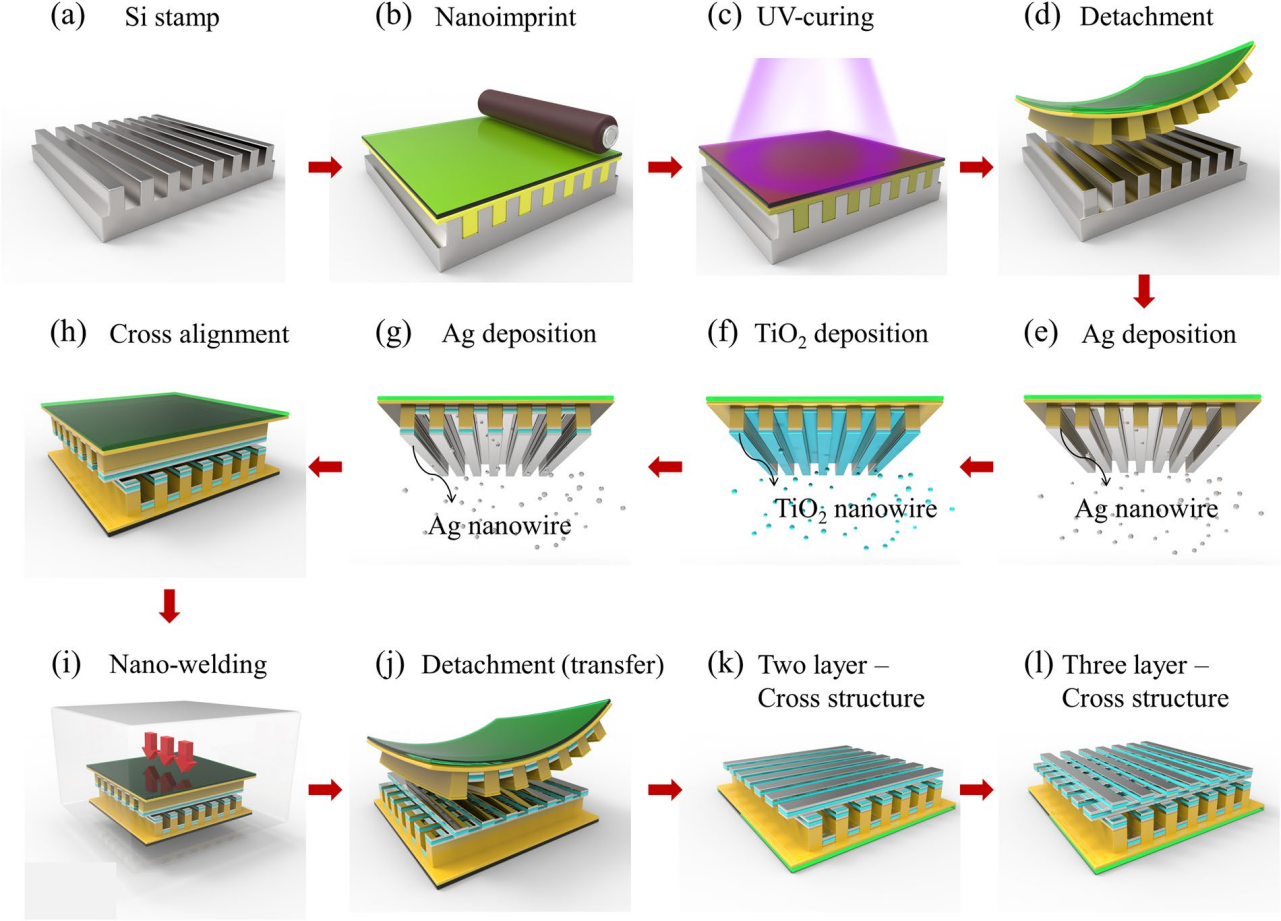
Schematic of fabrication process for Ag/TiO_2_ nanocomposite architecture: (**a**),(**b**) Formation process of nanocomposite wires with polymer pattern on PMMA film substrate based on nanoimprint lithography; (**c**),(**d**) UV-curing for 180 s and stamp detachment; (**e**)–(**g**) Formation of Ag/TiO_2_ nanocomposite wires by E-beam evaporator; (**h**),(**i**) Cross alignment and nanowelding process of Ag/TiO_2_ nanocomposite wires at the temperature of 90 °C, 0.5 MPa pressure, for 10 min under vacuum state; (**j**),(**k**) Detachment process and formation of Ag/TiO_2_ nanocomposite cross architecture with two layers; (**l**) Formation of Ag/TiO_2_ nanocomposite cross architecture with three layers by repeating process shown in (**i**),(**j**).

**Figure 3:**
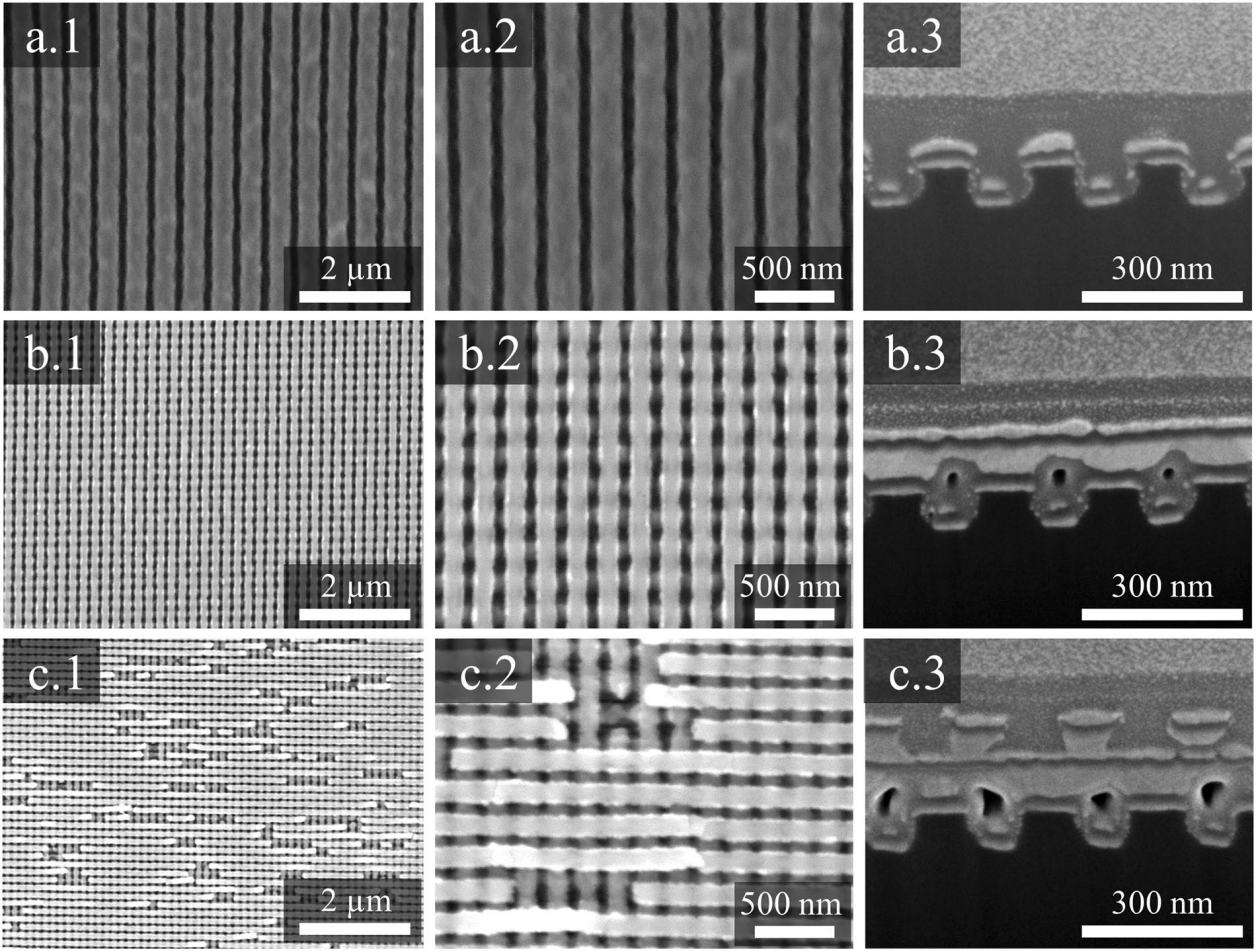
Top and cross-sectional SEM and FIB images of the fabricated samples: (**a**.**1**), (**a**.**2**), and (**a**.**3**) shows low-magnification, high-magnification, and cross-sectional images of Ag/TiO_2_ nanocomposite wires, respectively; (**b**.**1**), (**b**.**2**), and (**b**.**3**) show low-magnification, high-magnification, and cross-sectional images of two-layer Ag/TiO_2_ nanocomposite cross architecture, respectively; (**c**.**1**), (**c**.**2**), and (**c**.**3**) show low-magnification, high-magnification and cross-sectional images of three-layer Ag/TiO_2_ nanocomposite cross architecture, respectively.

Figure 2 shows the fabrication process for the Ag/TiO_2_ nanocomposite cross architectures. Facile evaporation using different materials based on layer-by-layer methods easily forms the nanocomposite wires architectures (see Fig. 2e–g). Ag/TiO_2_ nanocomposite wires are used to create Ag/TiO_2_ nanocomposite cross architectures with nanowelding and nanotransfer technologies (see Fig. 2h–k). By repeating the fabrication process shown in Fig. 2h–j, multi-layered nanocomposite cross architectures are formed (see Fig. 2i–l). The surface morphologies and cross-sectional images of the samples are shown in Fig. 3. The surface morphologies of the Ag/TiO_2_ nanocomposite wire architectures and the Ag/TiO_2_ nanocomposite cross architectures with two and three layers of nanocomposite wires are shown as Fig. 3a.1–2, b.1–2 and c.1–2, respectively. To better confirm the quality of the fabricated nanocomposite architectures, low-magnification images of the samples are obtained by SEM (see Fig. 3a.1–c.1). From Fig. 3a.1 and b.1, we can observe the fine surfaces of the Ag/TiO_2_ nanocomposite wire array and the Ag/TiO_2_ nanocomposite cross architecture, but defects appear on the surface of the three-layered Ag/TiO_2_ nanocomposite cross architecture because of the multiple transfers and welds of the thin Ag layers on the surface of the Ag/TiO_2_ nanocomposite wires during fabrication (see Fig. 3c.1). Cross-sectional images of the samples are shown in Fig. 3a.3–c.3. The Ag nanowire welding points in the Ag/TiO_2_ nanocomposite architecture surfaces are observed in the cross-sectional images (see Fig. 3b.3,c.3).

**Figure 4:**
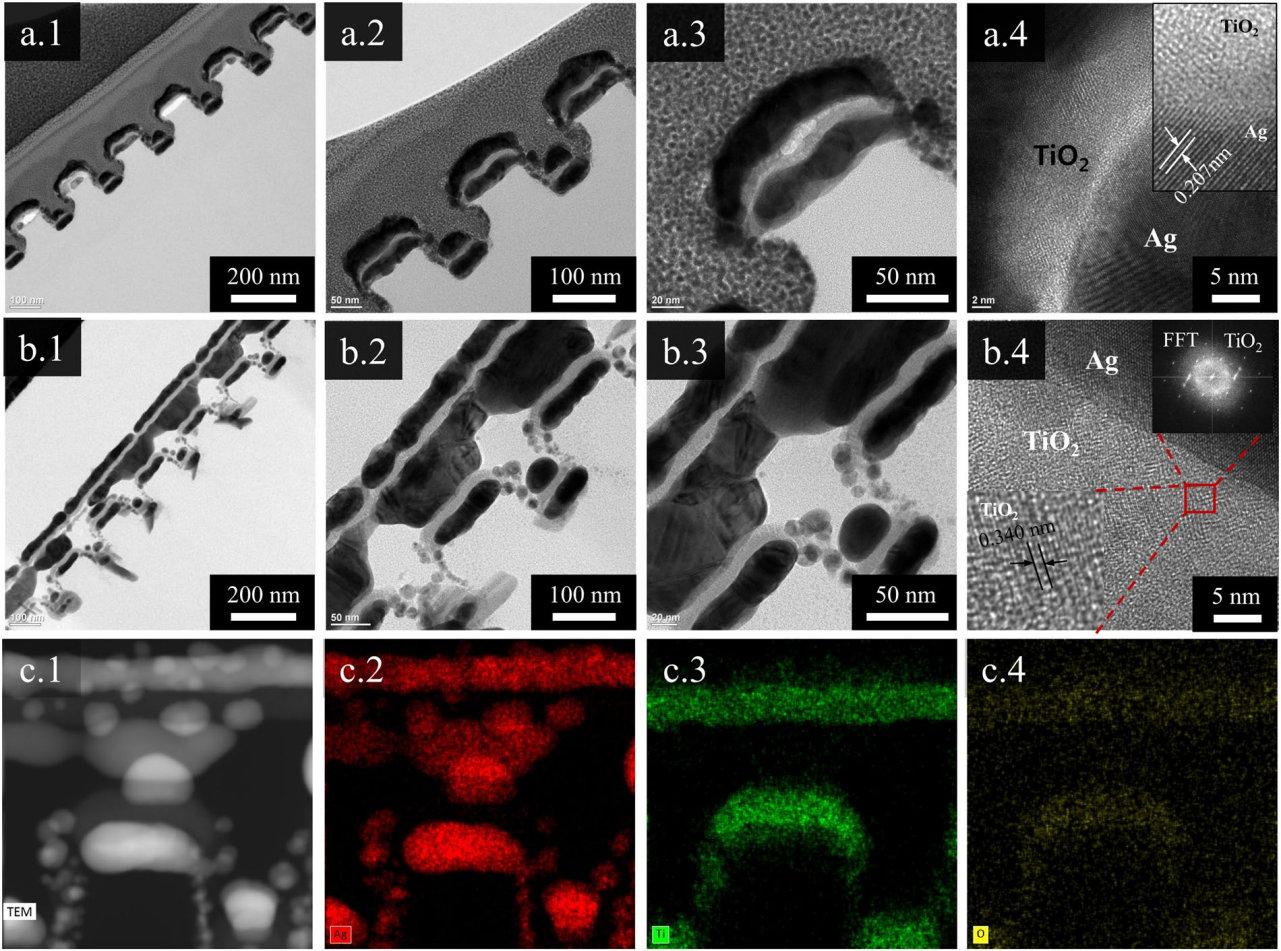
(**a**.**1**, **a**.**2**, and **a**.**3**) cross-sectional TEM images of Ag/TiO_2_ nanocomposite wires, (**a**.**4**) high-resolution TEM images in the Ag and TiO_2_ layers (inset: crystallinity of Ag and TiO_2_, scale: 1 nm); (**b**.**1**, **b**.**2**, and **b**.**3**) cross-sectional TEM image of two-layer Ag/TiO_2_ nanocomposite cross architecture, (**b**.**4**) high-resolution TEM image of Ag and TiO_2_ layers (insets: crystallinity and FFT image of TiO_2_). (**c**.**1**–**4**) TEM image of Ag/TiO_2_ nanocomposite cross architecture and the corresponding EDS mapping images of Ag, Ti, and O, in that order.

High-resolution TEM images of the fabricated samples were obtained to further investigate the recrystallization of welded Ag nanowires and the crystalline structures of the Ag and TiO_2_ nanocomposite wires. The TEM images of the Ag/TiO_2_ nanocomposite wires are shown in Fig. 4a. Well-aligned Ag/TiO_2_ nanocomposite wires on the polymer patterns are observed in Fig. 4a.1, a.2, and a.3. The lattice spacing is 0.207 nm (see Fig. 4a.4).

The interfacial crystalline structures of the Ag and TiO_2_ nanowires are shown in the inset of Fig. 4a.4. The fringe with a lattice space of 0.207 nm (Ag) is shown in the inset of Fig. 4a.4. Ag oxide is detected in very low quantities on the surface of the Ag nanowires. TEM images of the Ag/TiO_2_ nanocomposite cross architectures are displayed in Fig. 4b. Well-aligned nanocomposite cross architectures on the polymer patterns, an obvious interface between the Ag and TiO_2_ nanowires, and very precise welds between Ag and Ag nanowires on top of the Ag/TiO_2_ nanocomposite wires are observed in the TEM images (see Fig. 4b.1, b.2, and b.3). To better confirm the crystal structures of TiO_2_, the crystalline structures with clear fringe lattice of the TiO_2_ nanowires are clearly shown in the inset of Fig. 4b.4. The fringe lattice spacing of 0.340 nm appears in the TiO_2_ layer. In addition, fast Fourier transform (FFT) analysis was performed for the TiO_2_ crystal structures, and crystallization was observed via high-resolution TEM and compared with the FFT analysis. The FFT images of TiO_2_ are shown in the inset of Fig. 4b.4. Through the observations of clear fringe lattices and FFT images of TiO_2_, we can determine that TiO_2_ is crystal structures. In addition, the energy-dispersive X-ray spectrometry (EDS) mapping (Fig. 4c.1–4) suggests homogenous distributions of Ag, Ti, and O. Through the TEM observations, we can confirm that Ag/TiO_2_ nanocomposite wires and cross architectures were formed. In order to better demonstrate the formation of crystal structures, large-size high-resolution TEM images are provided as Supplementary Figure S3. However, the phase of TiO_2_ layer was still not confirm, so the Raman spectra was measured to analyse the specific peaks. The incident laser wavelength (λ = 514 nm) was chose to irradiate the fabricated sample. The Raman spectra of Ag/TiO_2_ nanocomposite cross architectures was shown in the Supplementary Figure S4. The weak peaks appeared at the 398 nm, 518 nm, and 630 nm wavelengths were observed and compared with previous studies^27–31^. We found that some peaks of TiO_2_ layer are consistent with the anatase TiO_2_. Therefore, we believe that the crystal structure of TiO_2_ layer is anatase.

### XPS spectral properties of the Ag/TiO_2_ nanocomposite architectures

**Figure 5:**
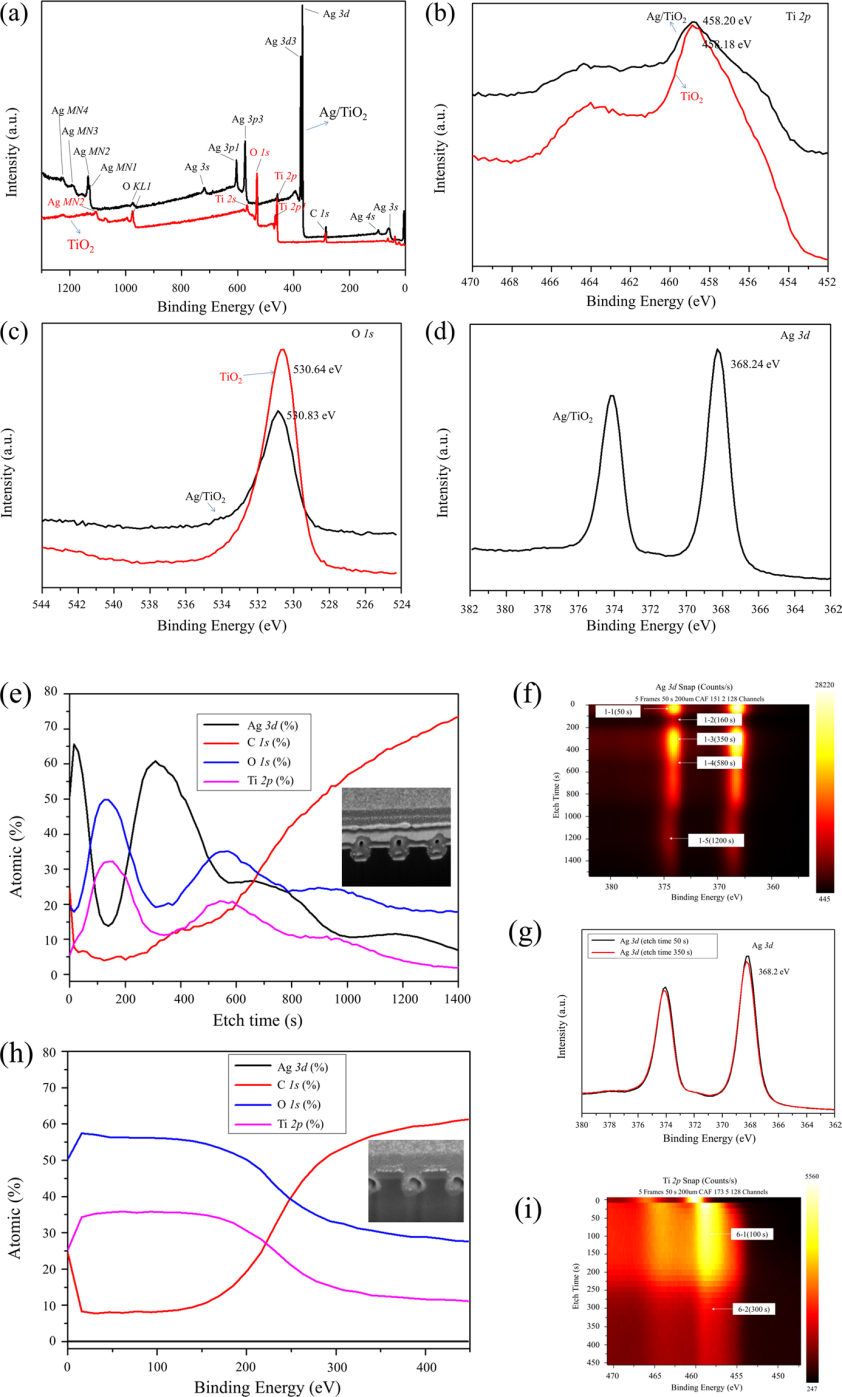
XPS spectra of two-layer Ag/TiO_2_ nanocomposite cross architecture and TiO_2_ nanowires: (**a**) survey spectra, (**b**) high-resolution Ti 2*p* spectra, (**c**) high-resolution O 1 *s* spectra, and (**d**) high-resolution Ag 3*d* spectrum of two-layer Ag/TiO_2_ nanocomposite cross architecture. (**e**) Atomic composition of two-layer Ag/TiO_2_ nanocomposite cross architecture depending on etching time. (**f**) Depth profile images of Ag and TiO_2_. (**g**) High-resolution Ag 3d spectra after etching for 50 s and 350 s. (**h**) Atomic composition of TiO_2_ nanowire depending on etching time. (**i**) Depth profile image of TiO_2_.

In order to understand the chemical components of the samples, the XPS spectra of the TiO_2_ nanowires and Ag/TiO_2_ nanocomposite cross architecture were analysed, as shown in Fig. 5. The scanned surface survey spectra illustrate the chemical composition of elemental Ti, O, and C in the TiO_2_ nanowires, and Ag, O, Ti, and C in the Ag/TiO_2_ nanocomposite cross architectures (see Fig. 5a). The high-resolution XPS spectra of Ag 3*d*, Ti 2*p*, and O 1 *s* are obtained to determine the chemical states of the elements and the interactions between the Ag and TiO_2_ nanowires (see Fig. 5b–d). Figure 5b shows the Ti 2*p* spectra for both the TiO_2_ nanowire and the Ag/TiO_2_ nanocomposite cross architecture, in which two peaks are observed at 458.2 eV and 458.18 eV. From the fabricated samples, the same values are observed for the Ti 2*p* peaks as those for pure TiO_2_, indicating the formation of a crystallized TiO_2_ layer embedded in the Ag nanowire layer. The binding energy of O 1 *s* (530.83 eV, attributed to elemental O in TiO_2_) is observed as shown Fig. 5c. Compared to the O 1 *s* spectrum of pure TiO_2_^32^, no significant changes or shifts are found. For the Ag 3*d* peaks of the Ag/TiO_2_ nanocomposite cross architecture, the binding energy is observed as shown in Fig. 5d. In comparison with the binding energy of bulk Ag, similar peaks are found. This indicates that the nanoarchitectures of the fabricated samples are effectively formed. To further observe the internal elements and the Ag welds in the fabricated samples, we chose an etching method to analyse the depth profile using a monoatomic Ar ion gun (energy of 500 eV, raster size of 1 × 1 mm, and angle of 30°). The etch process was implemented at intervals of 20 s for 80 cycles. In order to prevent the charging of samples, a flood gun with the pass energy of 151.8 eV was used for neutralizing. In addition, we used an X-ray beam of 200 µm and an Al Kα source to analyse and measure the fabricated samples. The depth profile analysis of the fabricated samples is shown in Fig. 5e–i. From Fig. 5e and h, the relative percentage of each chemical element can be observed, depending on the changes in etching time. Combined with Fig. 5e and f, we can determine the position of TiO_2_ layer and Ag layer welds at about ~160 s and 350 s, respectively. Figure 5g shows the Ag 3*d* peak at 50 s and 350 s; when the etch time is 50 s, the binding energy of the Ag nanowire as the top layer of the Ag/TiO_2_ nanocomposite cross architecture is shown, while at 350 s, the binding energy of the welds in the internal Ag nanowires is illustrated. From Fig. 5g, no change or shift is observed in the surface and welding positions of the Ag/TiO_2_ nanocomposite cross architecture. This means that the binding energy of Ag has no effect on the recrystallization of Ag during the fabrication of the Ag/TiO_2_ nanocomposite cross architecture. This result is consistent with the above TEM analyses. Figure 5h and i show the depth profile analyses of the TiO_2_ nanowires.

In order to obtain the exact compositions of the fabricated samples, we chose three etching times to find the exact compositions, as shown in Table 1. In addition, the composition of the Ag/TiO_2_ nanocomposite wire is shown in Supplementary Table S1, with corresponding XPS spectra in Supplementary Figure S5.

**Table 1:** The compositions of Ag/TiO_2_ nanocomposite cross architecture obtained by XPS at specified etching times.

Etch time (s)	Ag (%)	C (%)	O (%)	Ti (%)
160	17.20	5.04	46.83	30.93
580	26.16	18.51	34.51	20.82
1200	11.23	66.17	18.89	3.70

### Optical absorbance spectra of the Ag/TiO_2_ nanocomposite architectures

**Figure 6:**
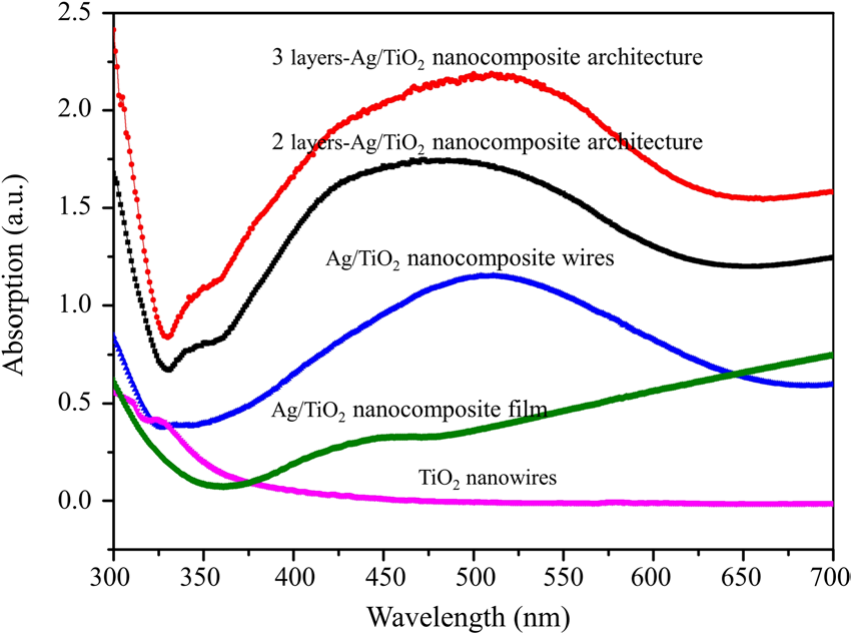
UV-vis absorption measurements of fabricated samples: three-layer Ag/TiO_2_ nanocomposites cross architecture, two-layer Ag/TiO_2_ nanocomposite cross architecture, Ag/TiO_2_ nanocomposite wires, Ag/TiO_2_ nanocomposite film, and TiO_2_ nanowires.

The optical absorbance spectra of the samples fabricated in this work are shown in Fig. 6. The absorption of the three-layered Ag/TiO_2_ nanocomposite cross architecture shows a maximum absorbance peak at λ = 510 nm, which is caused by the SPR phenomenon of the Ag layers embedded in the fabricated sample; this demonstrates that the surface defects in the architecture, shown in Fig. 3c.1, do not affect the SPR effects in the sample. In Fig. 6, the absorption spectra of three types of fabricated samples are shown; the maximum absorbance peak of each sample gradually weakens because of the weak SPR of the Ag layers embedded in the samples. The absorption spectrum of the two-layered Ag/TiO_2_ nanocomposite cross architecture shows a maximum absorbance peak at λ = 472 nm, while that of the Ag/TiO_2_ nanocomposite wires shows a maximum absorbance peak at λ = 511 nm. No peaks appear in the spectrum of the Ag/TiO_2_ nanocomposite film without any patterns, indicating that the interface of Ag and TiO_2_ does not experience the SPR phenomenon. Similarly, no absorption is shown for the TiO_2_ nanowires (Fig. 6); this is consistent with the abovementioned mechanism (under visible light, free electrons cannot be transferred to the conduction band from the valence band because of the 3.2-eV band gap of TiO_2_).

### Photocatalytic reaction efficiency and durability of the Ag/TiO_2_ nanocomposite architectures

By the abovementioned experiment, we confirmed that the photocatalytic reactions of Ag/TiO_2_ nanocomposite cross architectures are improved by their optical absorption based on SPR characteristics. In order to evaluate the photocatalytic activities of the fabricated samples, degradation experiments on MB solutions were performed under visible-light irradiation of 400–700 nm. The photocatalytic degradation of organic dyes and water pollutants is important in environmental pollutant treatment^17^.

**Figure 7:**
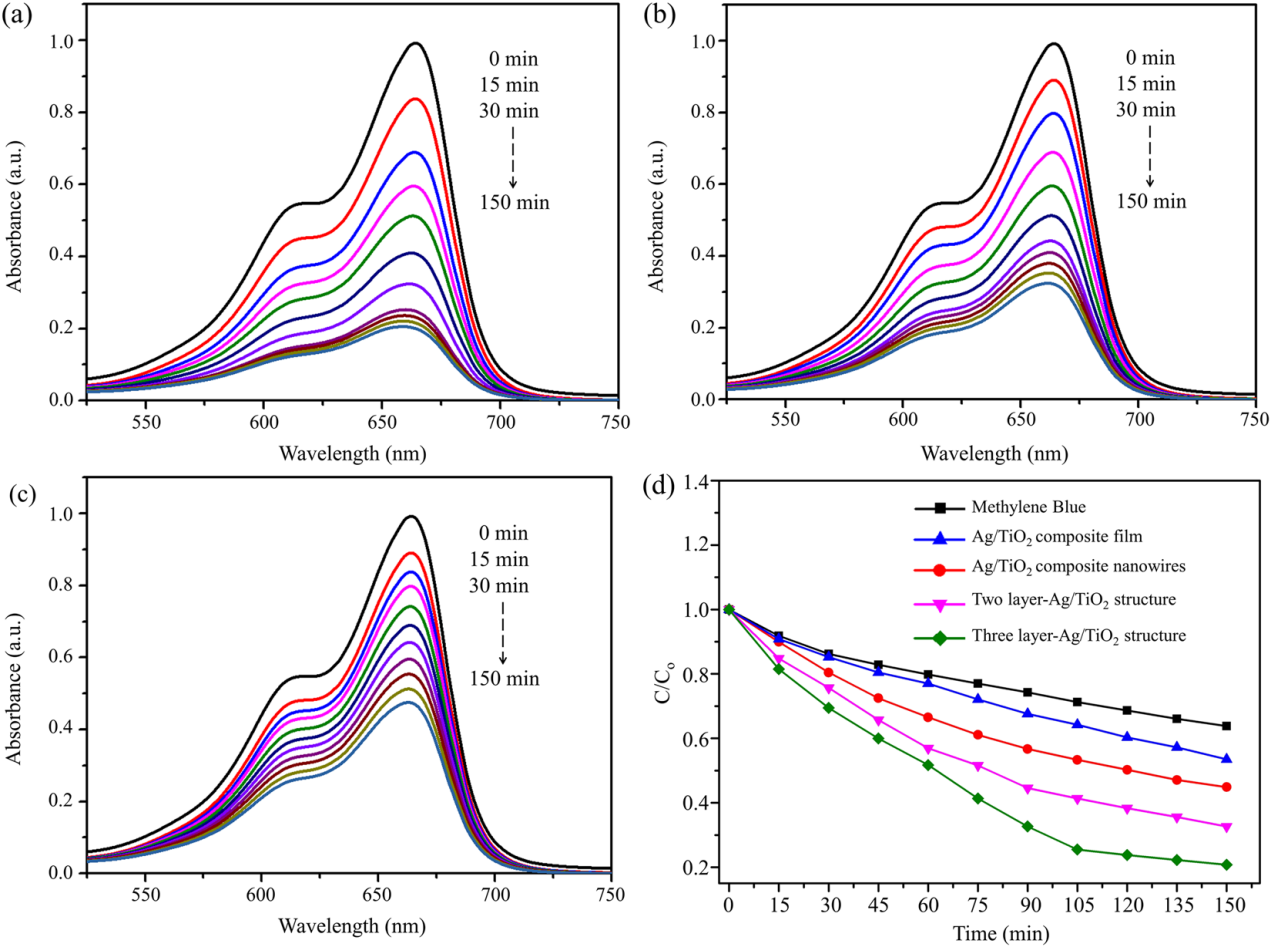
Photocatalytic reaction efficiency of the fabricated samples: (**a**) UV-vis absorbance spectra of MB with three-layer Ag/TiO_2_ nanocomposite cross architecture under visible-light irradiation every 15 min. (**b**) UV-vis absorbance spectra of MB with two-layer Ag/TiO_2_ nanocomposite cross architecture under visible-light irradiation every 15 min. (**c**) UV-vis absorbance spectra of MB with Ag/TiO_2_ nanocomposite wires under visible-light irradiation every 15 min. (**d**) Photocatalytic decomposition of MB solution with and without the fabricated samples under visible-light irradiation every 15 min (Ag/TiO_2_ nanocomposite wires, film, and two- and three-layer Ag/TiO_2_ nanocomposite cross architectures).

**Figure 8:**
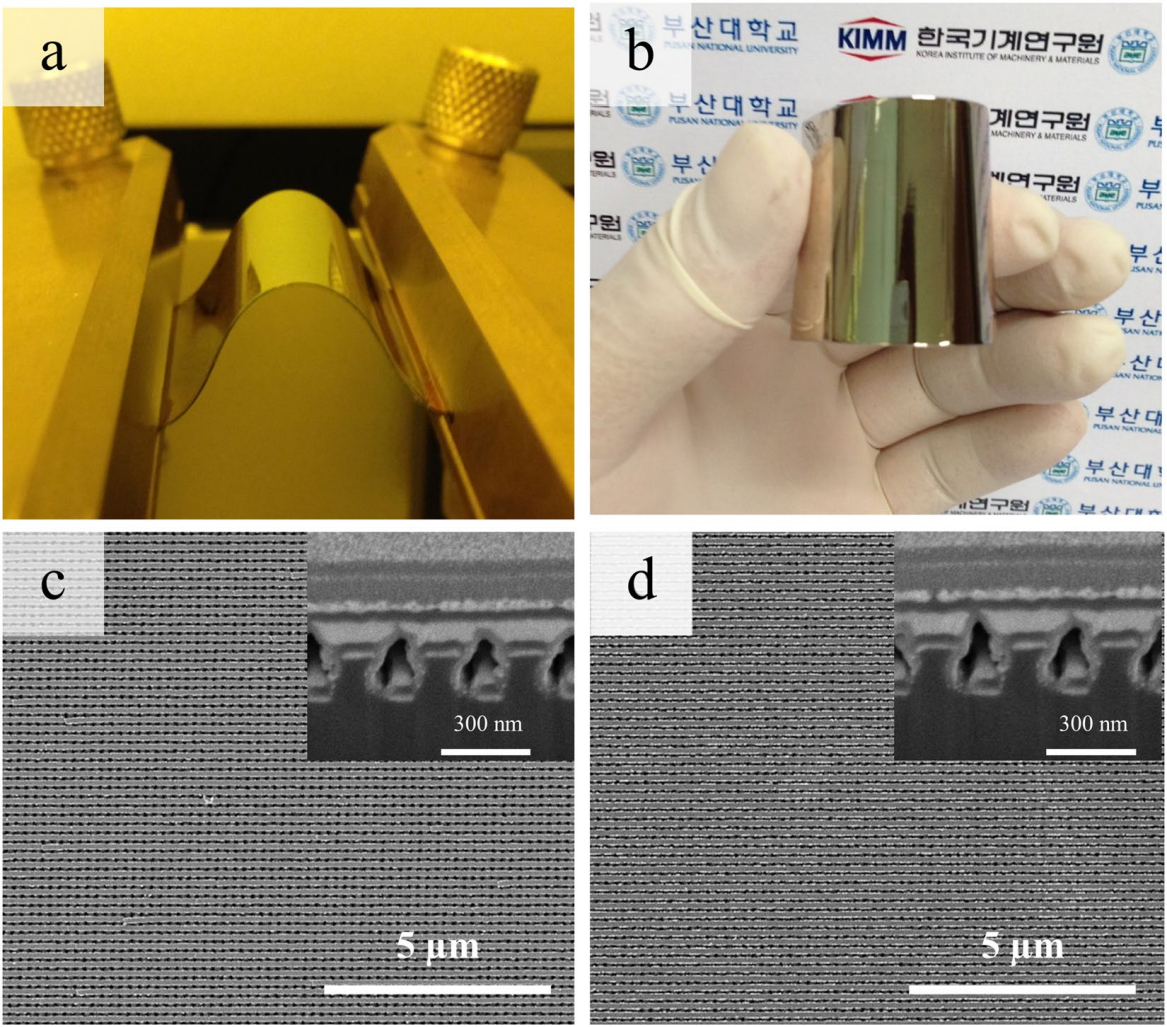
Photographs and SEM images of fabricated sample before and after bending test. (**a**) and (**b**) bending photographs; (**c**) and (**d**) SEM images of sample before and after bending test, respectively.

Figure 7a–c show the UV-vis absorbance spectra of MB at 15-min intervals of irradiation time using the Ag/TiO_2_ nanocomposite cross architectures with three and two layers, and the Ag/TiO_2_ nanocomposite wires alone, as photocatalysts, respectively. In addition, the UV-vis absorbance of MB at 15-min intervals of irradiation time for Ag/TiO_2_ nanocomposite film is shown in Supplementary Figure S2. The gradual degradation of MB is observed under visible-light irradiation. After 150 min of irradiation, 79.8% of MB is degraded by the three-layer Ag/TiO_2_ nanocomposite cross architecture, while 67.7% of MB is degraded by the two-layer architecture. Figure 7d shows the photocatalytic efficiency (C/C_0_) of the MB solution at λ = 663 nm after 0 to 150 min of visible-light irradiation on the various samples, where C is the absorbance of the MB solution after each 15-min irradiation interval and C_0_ is the initial absorbance of the solution under adsorption–desorption equilibrium with the fabricated samples, measured in darkness. The results indicate that the Ag/TiO_2_ nanocomposite cross architectures with three and two layers demonstrate higher photocatalytic efficiencies than the Ag/TiO_2_ nanocomposite wires under the same conditions, because of the stronger SPR phenomenon of multiple Ag layers under visible-light irradiation. The lower photocatalytic efficiency of the Ag/TiO_2_ nanocomposite wires and film is caused by the inefficient absorption of visible light, which is consistent with Fig. 6 and the mechanism shown in Fig. 1. However, the three-layered Ag/TiO_2_ nanocomposite cross architecture displays the highest photocatalytic efficiency among the samples because it has the largest surface area for SPR and broadband absorption. Through Figs 6 and 7, we demonstrate that the periodicity and number of layers of the 3D nanocomposite architectures significantly affect the SPR efficiency; thus, layered 3D nanocomposite architectures show enhanced photocatalytic activity. In order to evaluate the durability of the fabricated samples, bending tests were performed. Figure 8 shows photographs and SEM images of the fabricated sample before and after a cyclic bending test. Bending was performed with a radius of curvature of 1 cm for 15000 cycles (see Fig. 8a and b). The morphologies and cross-sectional images of the sample before and after the bending test are shown in Fig. 8c and d, respectively. By comparing their morphologies, we find neither cracks nor defects after the bending test for the fabricated 3D nanocomposite architecture. Therefore, we believe that simple bending tests can provide a good reference for the applicability of the photocatalyst in a curved container.

## Discussion

In summary, we proposed a new method to fabricate 3D nanocomposite architecture as a photocatalyst by using nanoimprint lithography, vertical e-beam evaporation, nano-transfer, and nanowelding to enhance photocatalytic activity by SPR. The proposed nanowelding technology easily overcame the fabrication difficulty of periodic 3D nanocomposite cross architectures, which exhibited strong SPR phenomena because of their large surface area and broadband light absorption. This phenomenon enhances photocatalytic activity on the surface of TiO_2_. The experiments showed that the 3D Ag/TiO_2_ nanocomposite cross architectures with three layers provided good photocatalytic performance, with the durability of the nanostructure demonstrated by cyclic bending tests. Flexible and efficient 3D nanocomposite architectures in photocatalysts fabricated in this method could be utilized in water disinfection, bacterial inactivation, and air purification.

## Methods

### Fabrication process for Ag/TiO_2_ nanocomposite architectures

The 3D nanocomposite architecture was fabricated using nanoimprinting, E-beam evaporation, nano-transfer, and nanowelding. First, Ormo-stamp resin (Micro Resist Technology, Germany) was used to coat a silicon stamp with 100-nm-wide/100-nm-spaced line patterns. Second, the stamp was covered with poly(methyl methacrylate) (PMMA) film and uniformly pressed by a nanoimprint roller, followed by curing with a UV light source (see Fig. 2a–c). Third, the PMMA film with polymer patterns was detached from the silicon stamp. One of the two prepared samples was treated with self-assembled monolayers (SAM), while the other was not (see Fig. 2d). Fourth, Ag and TiO_2_ were deposited on the prepared polymer patterns using a layer-by-layer method via an electron-beam (E-beam) evaporator (DAEKI HI-TECH Co, Ltd. Korea) (see Fig. 2e–g). For TiO_2_ deposition, we chose a Ta e-beam crucible to deposit because of the high melting point (1830 °C) of TiO_2_^33, 34^. Fifth, the Ag/TiO_2_ nanocomposite wires deposited on the polymer patterns with and without SAM treatment were aligned into cross architectures; afterward, heating nanowelding was performed by using thermal nanoimprinting (Hutem Co, Korea) at a temperature of 90 °C and a pressure of 0.5 MPa for 10 min (see Fig. 2h,i). The Ag/TiO_2_ nanocomposite wires deposited on the polymer patterns with SAM treatment, which weakened the adhesive force between the polymer pattern and deposited metal layers, were easily transferred to the Ag/TiO_2_ nanocomposite wires deposited on the non-SAM-treated polymer patterns, because of the stronger Ag welding on the Ag/TiO_2_ nanocomposite wire surfaces. Trichloro(1 H, 1 H, 2 H, 2H-perfluorooctyl)silane was selected as the SAM treatment material (Sigma-Aldrich)^35, 36^. One of the PMMA films with polymer patterns was detached from the welded nanocomposite cross architecture (see Fig. 2j,k). By repeating the fabrication process shown in Fig 2h–j and 3D nanocomposite architectures were fabricated (see Fig. 2i–l). In order to compare with the photocatalytic activity of the 3D nanocomposite architectures, a Ag/TiO_2_ nanocomposite film without any pattern was fabricated on the PMMA substrate based on the layer-by-layer method via e-beam vertical evaporation under high vacuum of 6 × 10^−6^ Torr, the constant rate of 1.0 Å/s, and a certain rotation rate. The voltage and current were controlled at approximately 6.35 V and 120 mA, respectively. The morphology and cross-sectional images were shown in Supplementary Figure S1.

### Characterization

In order to investigate various characteristics of the fabricated samples, a field-emission scanning electron microscope (FE-SEM; Sirion, FEI Netherlands), focused ion beam system (FIB; Helios Nanolab, FEI Netherlands), and transmission electron microscope (TEM; JEM-ARM200F, JEOL Japan) were used to observe the surface morphologies and cross-sectional images of the structures. The compositions of the 3D nanocomposite cross architectures were analysed by using an X-ray photoelectron spectrometer (XPS) (K-Alpha+, Thermo Fisher Scientific, Inc.). The crystalline structures and atomic arrangements of the Ag/TiO_2_ nanocomposite wires and cross architectures were analysed via TEM. The phase of TiO_2_ layer was analysed and compared by using high resolution Raman System (LabRAM HR Evolution Visible NIR, Horiba). The optical absorptions of the fabricated samples were measured to compare their properties using a UV-vis spectrometer (S-3100, Analysis Measuring, Scinco). In addition, the photocatalytic degradation of MB was performed to evaluate the photocatalytic effect of the Ag/TiO_2_ nanocomposite cross architectures. The various fabricated samples measuring 3 × 3 cm were immersed into 5 mL of 4-ppm MB aqueous solution before exposure to an Xe light source (Avalight-LDXE, Avantes BV), having a 600-µm fibre operating at 153 mW, a visible-light filter for wavelengths of ~400–700 nm, and an optical lens. The available wavelength range of visible light was ~400–685 nm because of optical filter losses. The irradiation was performed at 15-min intervals. Simultaneously, the absorptions of the decomposed MB solution were measured using the UV-vis spectrometer to evaluate the photocatalytic decomposition of the solution.

## Electronic supplementary material

Supplementary information
