# Supplementary material for: Three-dimensional plasmonic Ag/TiO2 nanocomposite architectures on flexible substrates for visible-light photocatalytic activity
Source: Sci Rep. 2017 Aug 21;7:8915. doi: 10.1038/s41598-017-09401-z (PMC5566718; doi:10.1038/s41598-017-09401-z)
Supplement: Supplementary file 1 — Supplementary information [file 41598_2017_9401_MOESM1_ESM.doc]

Three-dimensional plasmonic Ag/TiO_2_ nanocomposite architectures on flexible substrates for visible-light photocatalytic activity

*Zhi-Jun Zhao^1,2^, Soon Hyoung Hwang^2,3^, Sohee Jeon^2^, Boyeon Hwang^2,4^, Joo-Yun Jung^2^, Jihye Lee^2^, Sang-Hu Park^1^*, and Jun-Ho Jeong^2^**

^1^School of Mechanical Engineering, Pusan National University, Busandaehak-ro 63beon-gil, Geumjeong-gu, Busan 609-735, Republic of Korea

^2^Department of Nano Manufacturing Technology, Korea Institute of Machinery and Materials, Daejeon 305-343, South Korea

^3^Research Institute of Advanced Materials (RIAM), Department of Materials Science and Engineering, Seoul National University, Daehak-Dong, Gwanak-Gu, Seoul 151-744, Korea

^4^School of Electrical Engineering, Collage of Engineering, Korea University, Seoul, 02841, Republic of Korea

* Corresponding authors:

Prof. Sang-Hu Park

Tel.: +82-51-510-1011; Fax: +82-51-514-0685

E-mail: sanghu@pusan.ac.kr

Dr. Jun-Ho Jeong

Tel.: +82-42-868-7604; Fax: +82-42-868-7123

E-mail: jhjeong@kimm.re.kr


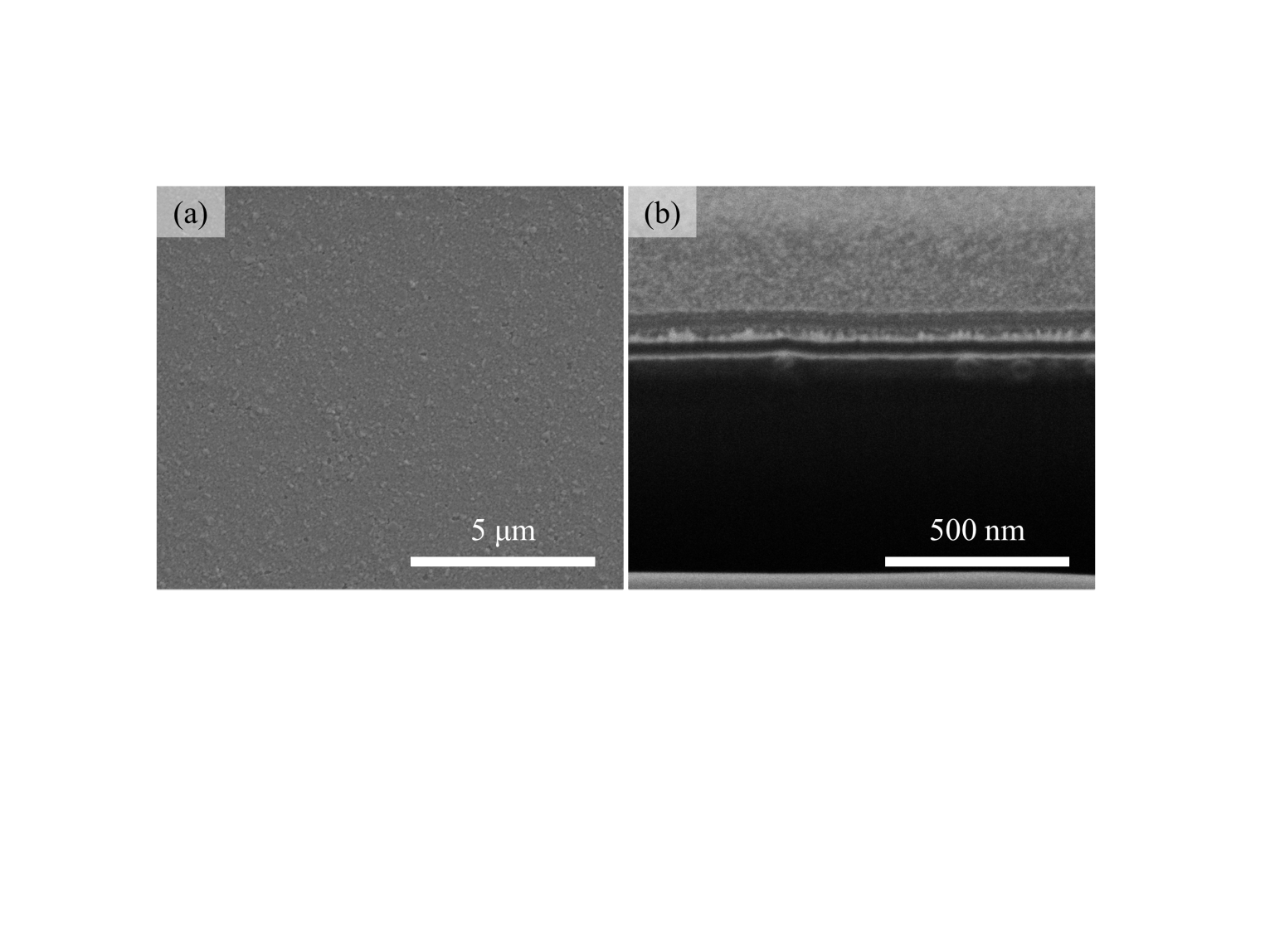


Figure S1. The morphology (a) and cross-sectional image (b) of Ag/TiO_2_ nanocomposite film without any patterns.


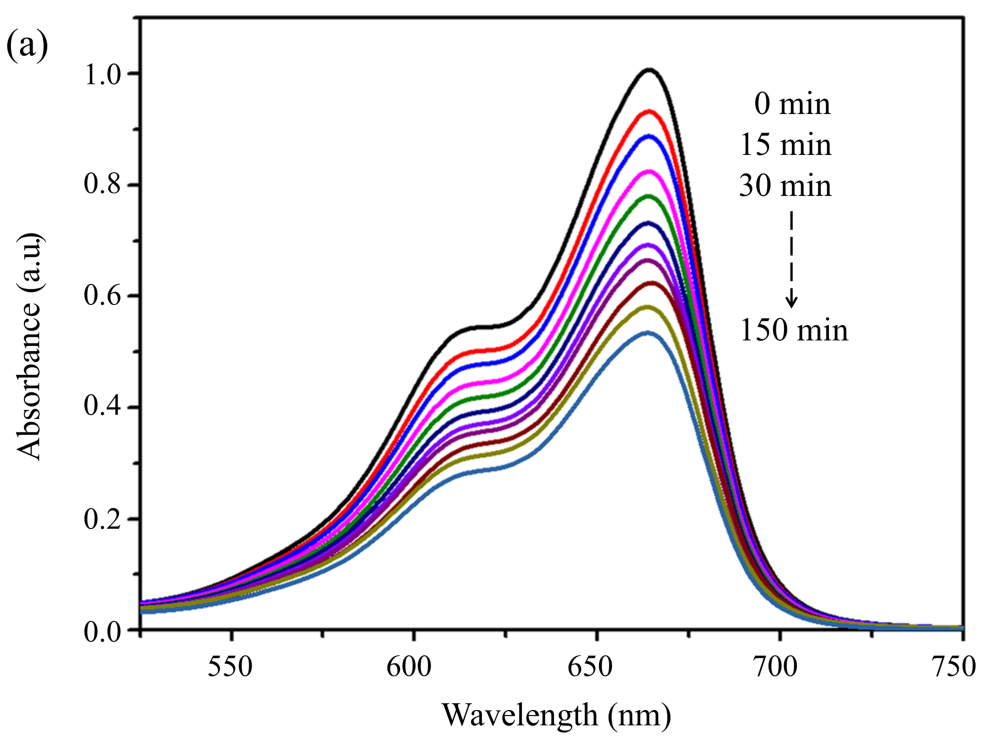


Figure S2. UV-vis absorbance spectra of MB with Ag/TiO_2_ nanocomposite film (without any patterns) under visible-light irradiation every 15 min.

**
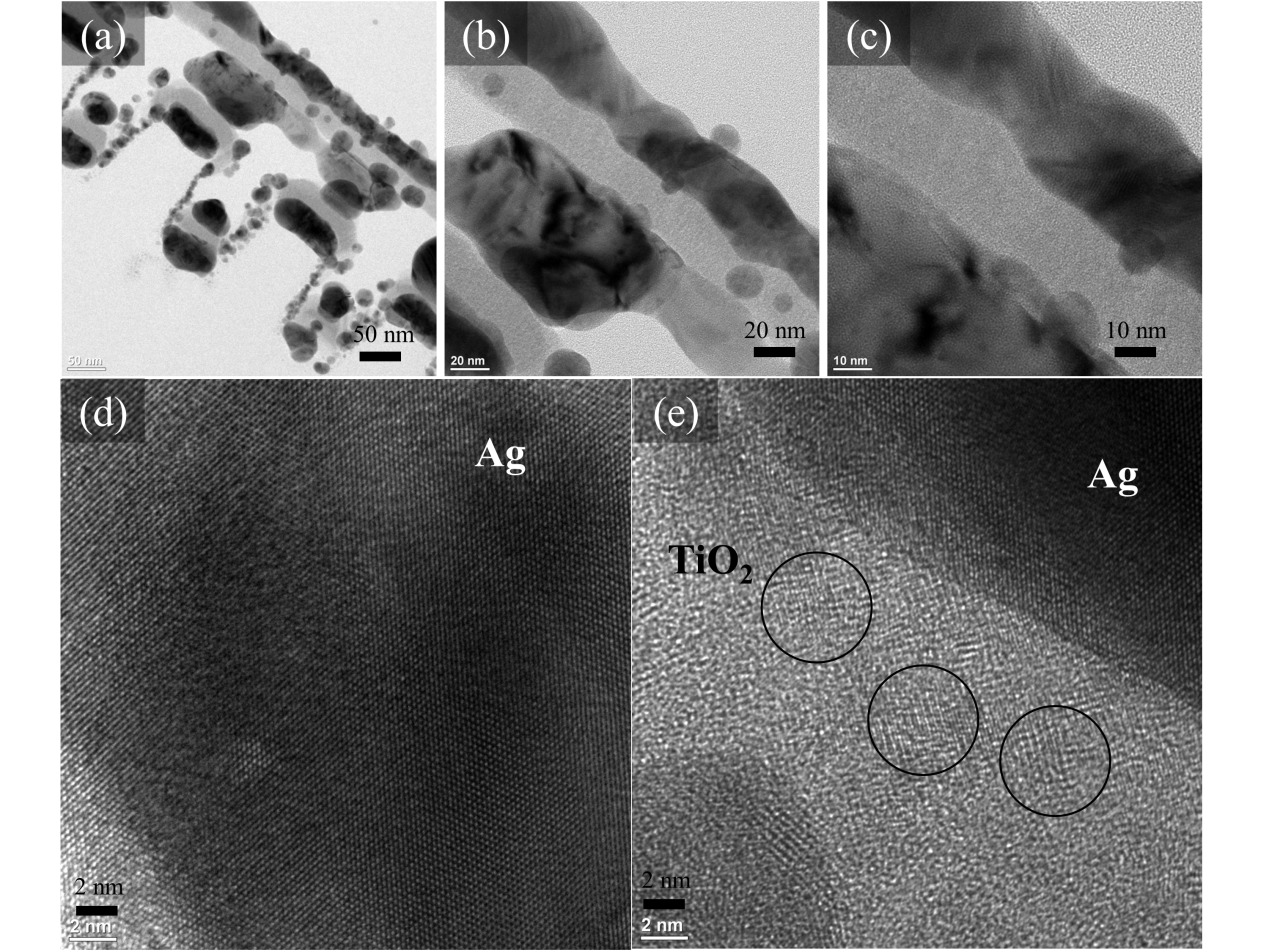
**

Figure S3. (a-c) high-magnification cross-sectional TEM images, (d) crystal structure of Ag, (e) crystal structure of TiO_2_ layer

| **Etch time (s)** | **Ag (%)** | **C (%)** | **O (%)** | **Ti (%)** |
| --- | --- | --- | --- | --- |
| 46 | 72.06 | 2.80 | 15.81 | 9.34 |
| 123 | 8.97 | 2.77 | 53.49 | 34.77 |
| 200 | 11.23 | 66.17 | 18.89 | 3.70 |
| 308 | 32.35 | 2.10 | 38.18 | 27.37 |
| 386 | 63.76 | 13.72 | 14.35 | 8.17 |

Table S1. The composition of Ag/TiO_2_ nanocomposite nanowires obtained by XPS at specific etching times


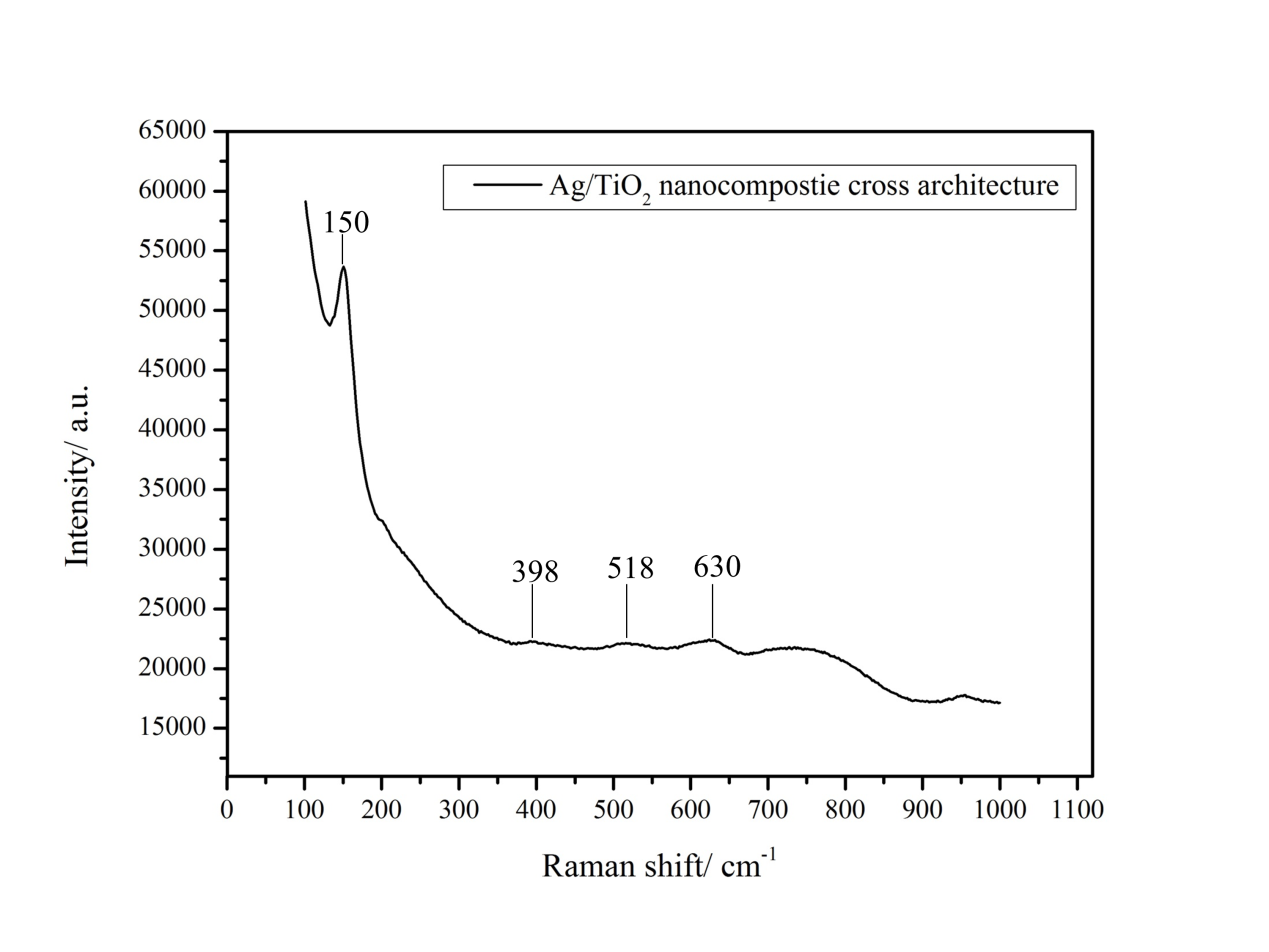


Figure S4. Raman spectra of the Ag/TiO_2_ nanocomposite cross architectures.


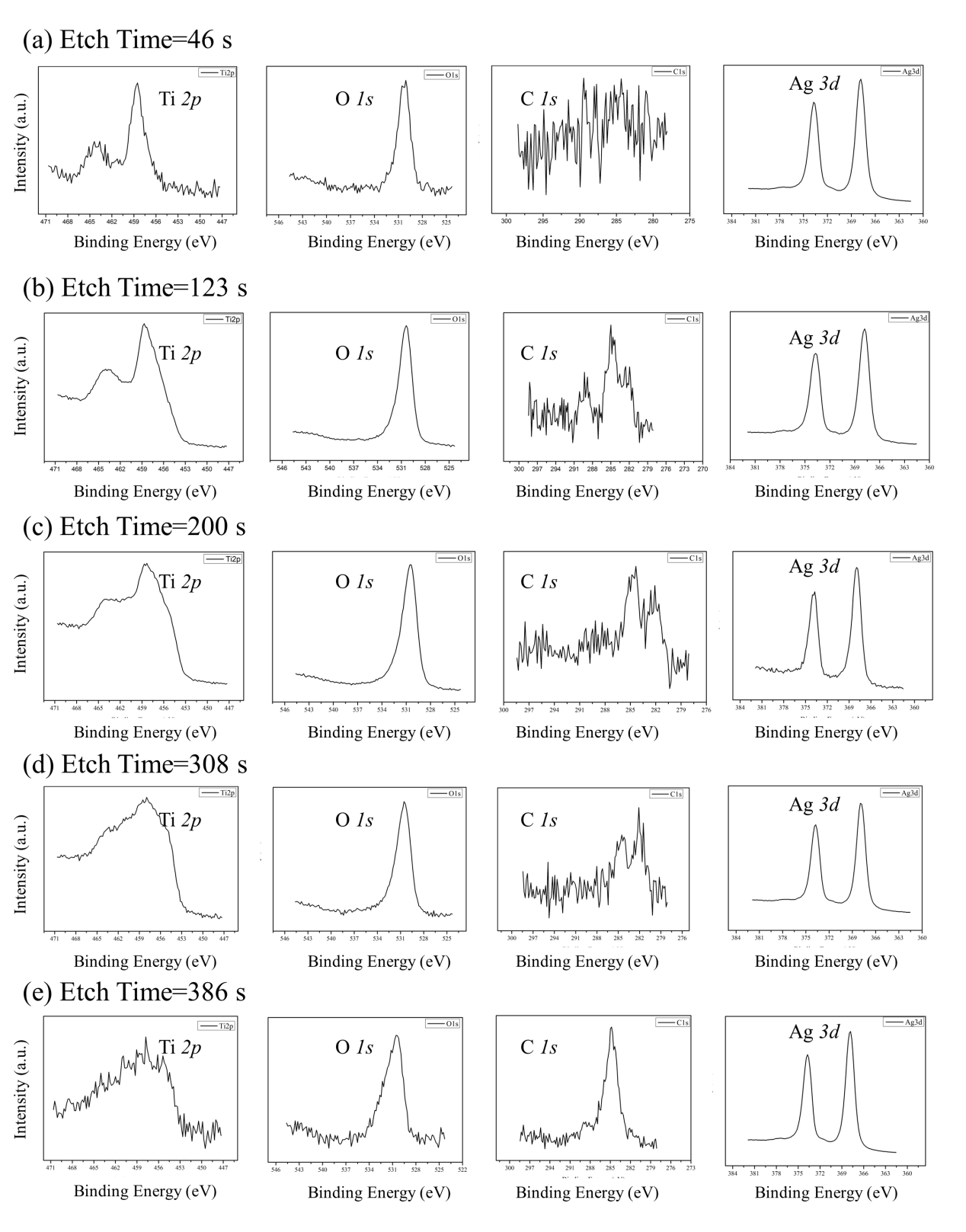


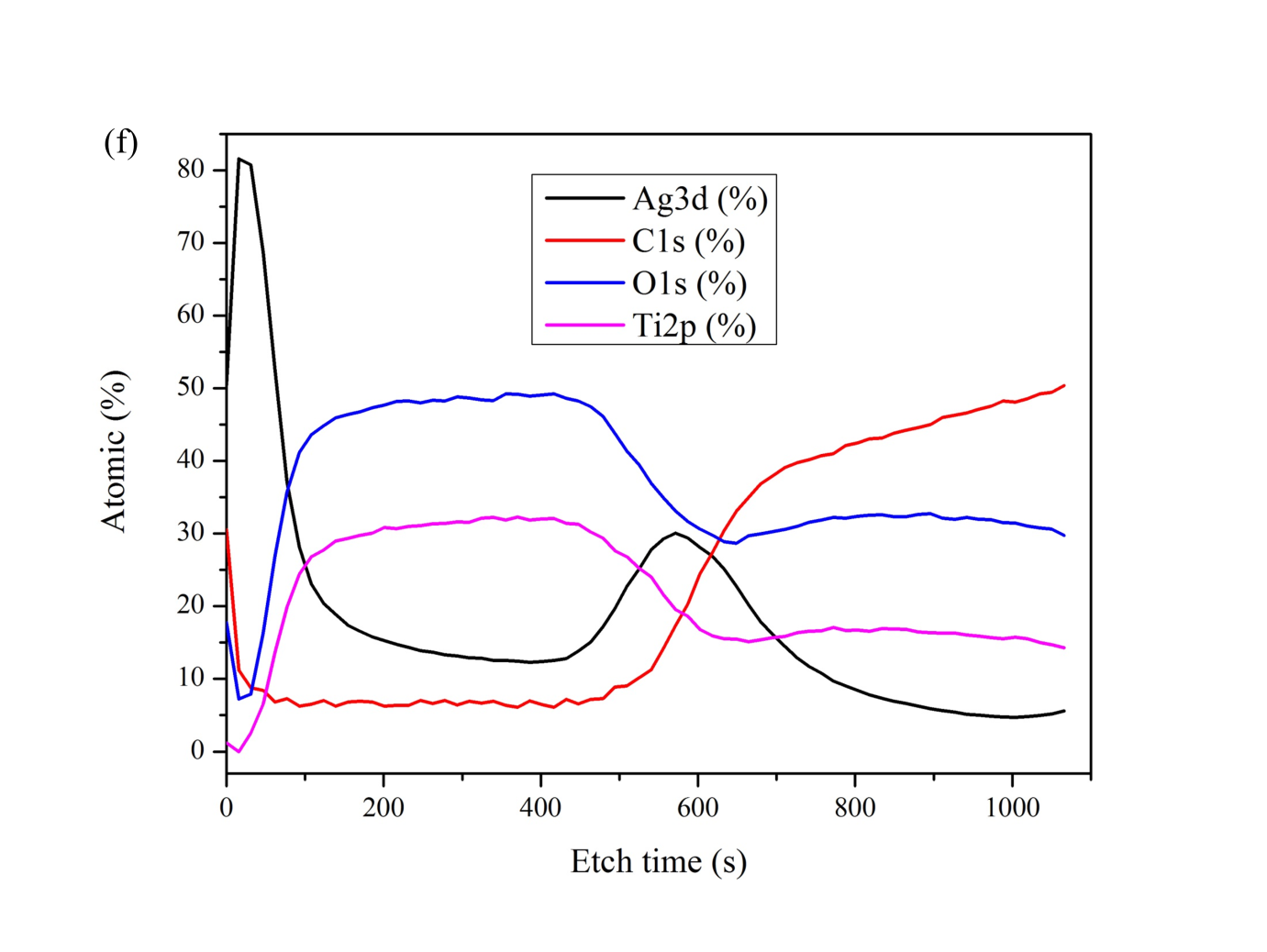


Figure S5. XPS spectra of the Ag/TiO_2_ nanocomposite nanowires depending on the etch time (a) 46 s, (b) 123 s, (c) 200 s, (d) 308 s, (e) 386 s. (f) Atomic composition.
